# Pathology and Medicine

**Published:** 1858-03

**Authors:** 


### PATHOLOGY AND MEDICINE.

1. Liability of Negroes to the Endemic Diseases of the South.—The fact is so glaring, and so universally admitted, that I am really at a loss to select evidence to show that there is no acclimation against the endemic fevers of our rural districts. Is it not the constant theme of the population of the South, how they can preserve health ? and do not all prudent persons, who can afford to do so, remove in the summer to some salubrious locality, in the pine-lands or the mountains ? Those of the tenth generation are just as solicitous on the subject as those of the first. Books written at the North talk much about acclimation at the South ; but we here never hear it alluded to out of the yellow-fever cities. On the contrary, we know that those who live from generation to generation in malarious districts become thoroughly poisoned, and exhibit the thousand Protean forms of disease which spring from this insidious poison.

I have been the examining physician to several life-insurance companies for many years, and one of the questions now asked in many of the policies is, "Is the party acclimated ?" If the subject lives in one of our southern seaports, where yellow fever prevails, and has been born and reared there, or has had an attack of yellow fever, I answer, "Yes." If, on the other hand, he lives in the country, I answer, "No ;" because there is no acclimation against intermittent and bilious fevers, and other marsh diseases. Now, I ask if there is an experienced and observing physician at the South who will answer differently ? An attack of yellow fever does not protect against marsh fevers, nor *vice versa*.

The acclimation of negroes, even, according to my observation, has been put in too strong a light. Being originally natives of hot climates, they require no acclimation to temperature, are less liable to the more inflammatory forms of malarial fevers, and suffer infinitely less than whites from yellow fever: they never, however, as far as my observation extends, become proof against intermittents and their sequelæ. The cotton planters throughout the South will bear witness, that, wherever the whites are attacked with intermittents, the blacks are also susceptible, though not in so great a degree. My observations apply to the region of country removed from the rice country. We shall see, further on, that the negroes of the rice-field region do undergo a higher degree of acclimation than those of the hilly lands of the interior. I know many plantations in the interior of Alabama, South Carolina, Georgia, Mississippi, and Louisiana, on which negroes of the second and third generation continue to suffer from these malarial diseases, and where gangs of negroes do not increase. (P. 376.)

And again: Certainly, negroes do suffer greatly on many cotton plantations in the middle belt of the Southern States; and I have seen no evidence to prove that negroes can, in this region, become accustomed to the marsh poison; and my observation has been extensive in four States. A question here arises: Is their any difference in types of those malarial fevers which originate in the flat tide-water rice-lands, and those of the clay-hills, or marsh fevers of the interior? I am inclined to think there is.—*Indigenous Races*, p. 376; Art. "Acclimation," etc. By J. C. NOTT, M.D., of Mobile, Alabama.

2. CLINICAL OBSERVATIONS ON EPILEPSY. By *Dr. Th. Herpin.*—Dr. Herpin's name is familiar to the medical profession on account of his urgent advocacy of the employment of oxide of zinc in the treatment of epilepsy. He now abandons this preparation entirely in favor of the lactate of zinc. The greater solubility and digestibility of the latter would *à priori* engage our sympathies in its favor. The following is one of the cases which have decided the author's preference, and deserves to be recorded on account of the success obtained in spite of the unfavorable prognosis which the case would have justified in the first instance.

Miss E., aged eleven and a half, consulted Dr. Herpin, February 1st, 1854. She was well made, intelligent, and pleasing. Her paternal grandfather had died epileptic at forty-nine years of age; her maternal grandfather at seventy-six, after being insane for six years, and in a state of melancholy for forty years. No predisposing or exciting cause was traceable in the patient, excepting perhaps the fright caused by a fire two months previous to the first attack. She enjoyed excellent health till six years of age, when she had typhoid fever, having on the right side a tendency to slight and evanescent deafness. Three months before the first seizure she was attacked with frequeut headache, generally commencing in the morning, and lasting till evening. The headache ceased in December, 1853. The first fit occurred on August 15th, 1853, the second on September 5th, the third on September 26th; six then followed at variable intervals, making altogether nine in less than five months; they always took place during the first hour of sleep, and the evening before the attack she was observed to be somewhat excited. The symptoms, which are detailed, leave no doubt as to the attacks having been those of genuine epilepsy. The oxide of zinc had been prescribed for the patient from September to the following July, but she was unable to bear the doses which Dr. Herpin thinks necessary in order to make a proper impression upon the patient. He was once able to reach a dose of six grammes (ninety grains) per week, but he was obliged to diminish it. Still the attacks ceased from January the 8th to July the 18th; but a return on that day, brought on by a tepid bath, induced Dr. Herpin to have recourse to the lactate of zinc, which he gave for above six months, during which time the patient swallowed 306 grammes, (4600 grains.) The tolerance of the remedy was complete, and when she left off taking it she was in perfect health. There was one recurrence of epilepsy two months after commencing the lactate. Three years have since elapsed, and the lady's health continues sound.—*L' Union Médicale*, No. 121, 1857. *British and Foreign Med.-Chir. Review*, January, 1858.

